# Paper-based MoS_2_ nanosheet-mediated FRET aptasensor for rapid malaria diagnosis

**DOI:** 10.1038/s41598-017-17616-3

**Published:** 2017-12-13

**Authors:** Alisha Geldert, Kenry, Chwee Teck Lim

**Affiliations:** 1 Department of Biomedical Engineering, National University of Singapore, Singapore, 117576 Singapore; 2 NUS Graduate School for Integrative Sciences and Engineering, National University of Singapore, Singapore, 117456 Singapore; 3 Centre for Advanced 2D Materials and Graphene Research Centre, National University of Singapore, Singapore, 117543 Singapore; 4 Mechanobiology Institute, National University of Singapore, Singapore, 117411 Singapore

## Abstract

There has been growing interest in the development of paper-based biosensors because their simplicity and low cost are attractive for point-of-care diagnosis, especially in low-resource areas. However, only a limited range of paper materials – primarily chromatography papers – have been incorporated into diagnostics thus far. Here, we investigate the performance of different types of paper in order to develop an aptamer- and MoS_2_ nanosheet-based sensor relying on fluorescence resonance energy transfer (FRET) to signal the presence of a target protein. An aptamer which binds to a malarial biomarker, *Plasmodium* lactate dehydrogenase (pLDH), is chosen for this study, as point-of-care diagnostics would be especially advantageous in low-resource areas, such as those where malaria is prevalent. We observe that of all papers tested, a measurable and specific fluorescence recovery can only be produced on the sensor created with printer paper, while no significant fluorescence recovery is generated on sensors made from other types of paper, including chromatography, lens, and filter papers. Therefore, our findings demonstrate the importance of careful material selection for the development of a paper-based diagnostic test, and suggest that commercially-available products such as printer paper may serve as viable materials to develop cost-effective and simple diagnostics.

## Introduction

Access to rapid and accurate disease diagnosis is an important aspect of global health efforts. Proper diagnosis is necessary not only to identify patients requiring medical care, but also to discriminate between diseases which manifest a similar set of symptoms. For instance, many tropical diseases such as malaria and dengue have similar clinical presentations, but must be differentiated to prevent prescribing unnecessary or improper drugs, which may lead to further aggravation or drug resistance^1^. However, many low-resource areas lack the equipment, infrastructure, or expertise necessary to diagnose diseases using standard methods. Recently, there has been growing interest in developing low-cost and portable point-of-care diagnostic tools, such as dipstick^2,3^, lateral flow^4^, or microfluidic devices^5^ to address this issue.

Paper has been increasingly used in point-of-care diagnostics due to its low cost, wide availability, portability and small storage volume. Most paper-based assays use chromatography paper because of its uniformity and wicking ability, which enable a sample to be transported through the paper to a reaction zone. Test results are usually indicated by a color change or other visible readout in the reaction zone^6^. Definitive assessment of colorimetric assays can sometimes be challenging due to inhomogeneous color distribution on the test strip^7^ or changes which are indistinguishable to the naked eye^8^. Paper-based fluorescent assays may offer more quantitative results with improved sensitivity, but have only just begun to be explored. One of the first examples of fluorescence measurements from paper was demonstrated recently using patterned paper microzone plates from which the fluorescence or absorbance of many reaction zones could be measured at once using a standard microplate reader^9^. More portable and simple fluorescence readers suitable for point-of-care use have also been developed recently using materials such as LEDs, photodiodes, and plastic filters^10,11^. With the development of simpler and more affordable fluorescence assays and readers, fluorescence-based detection may become a viable sensing strategy for point-of-care diagnostics.

A facile fluorescence-based detection scheme with potential applications in low-resource areas is aptamer-based sensing (i.e., aptasensing). Aptamers are short, single-stranded nucleic acids which can be developed to bind specifically to various biomolecular targets^12^. As compared to antibodies, which are commonly used in rapid diagnostic tests, aptamers have similar affinity to their targets but are simpler and cheaper to produce and are more stable in harsh conditions, making them attractive for use in point-of-care diagnostics^13,14^. Most aptasensors operate based on the principle of fluorescence resonance energy transfer (FRET), in which fluorescently-labeled aptamers are released from a fluorescence quenching material upon binding to their targets. This release induces a measurable increase in fluorescence to indicate the presence of target biomolecules in an analyte. FRET-based aptasensors with nanomolar or even picomolar limits of detection have been developed for many biomolecules of interest, including amyloid β oligomers^15^, single-stranded DNA^16^, insulin^17^, platelet-derived growth factor^18^, thrombin^16^, and a malarial lactate dehydrogenase^19,20^. However, most of these sensors are liquid-based, in which solutions of aptamers, quenchers, and an analyte containing the target molecules must be mixed together before detection proceeds. This strategy requires large reagent volumes and multiple assay steps, which would make it challenging to be applied in low-resource areas. Paper-based sensing could offer a simpler and more portable alternative; however, the few paper-based FRET aptasensors which have been developed thus far often require complex assay procedures, higher aptamer or quencher concentrations, or fluorescence imaging^21–23^. However, with further simplification and optimization, paper-based FRET aptasensors could offer a more robust mechanism of diagnosis.

While chromatography paper has been the standard substrate for paper-based colorimetric assays, there has been little investigation into the optimal type of paper for alternative sensing schemes such as FRET-based aptasensing. In fact, the choice of paper may have a strong influence on sensor sensitivity and specificity. For instance, a recent study reported that paper thickness and density significantly affected the performance of a paper-based blood typing assay^24^. In general, however, few studies have compared the performance of different types of paper in any sort of diagnostic assay even though the hydrophilicity, pore size and porosity, surface area, thickness, fiber structure, and grammage of different paper materials can vary widely. Investigation of more affordable and accessible commercially-available paper products such as coffee filters, tissues, or printing paper could also be advantageous for diagnostics, but these forms of paper have rarely been considered for use in biosensors.

As such, we evaluate the use of several types of paper, including both commercially-available and scientific-grade products, for the development of a FRET-based paper aptasensor for the detection of a malarial biomarker, *Plasmodium* lactate dehydrogenase (pLDH). To the best of our knowledge, this is the first study evaluating how different types of paper would affect FRET-based aptasensor performance as well as the first use of commercially-available paper for FRET-based aptasensing. We anticipate that this work will demonstrate important differences between several potential materials for paper-based diagnostics, and further contribute to the development of a facile, disposable, and low-cost diagnostic scheme for malaria and other diseases.

## Results and Discussion

**Figure 1:**
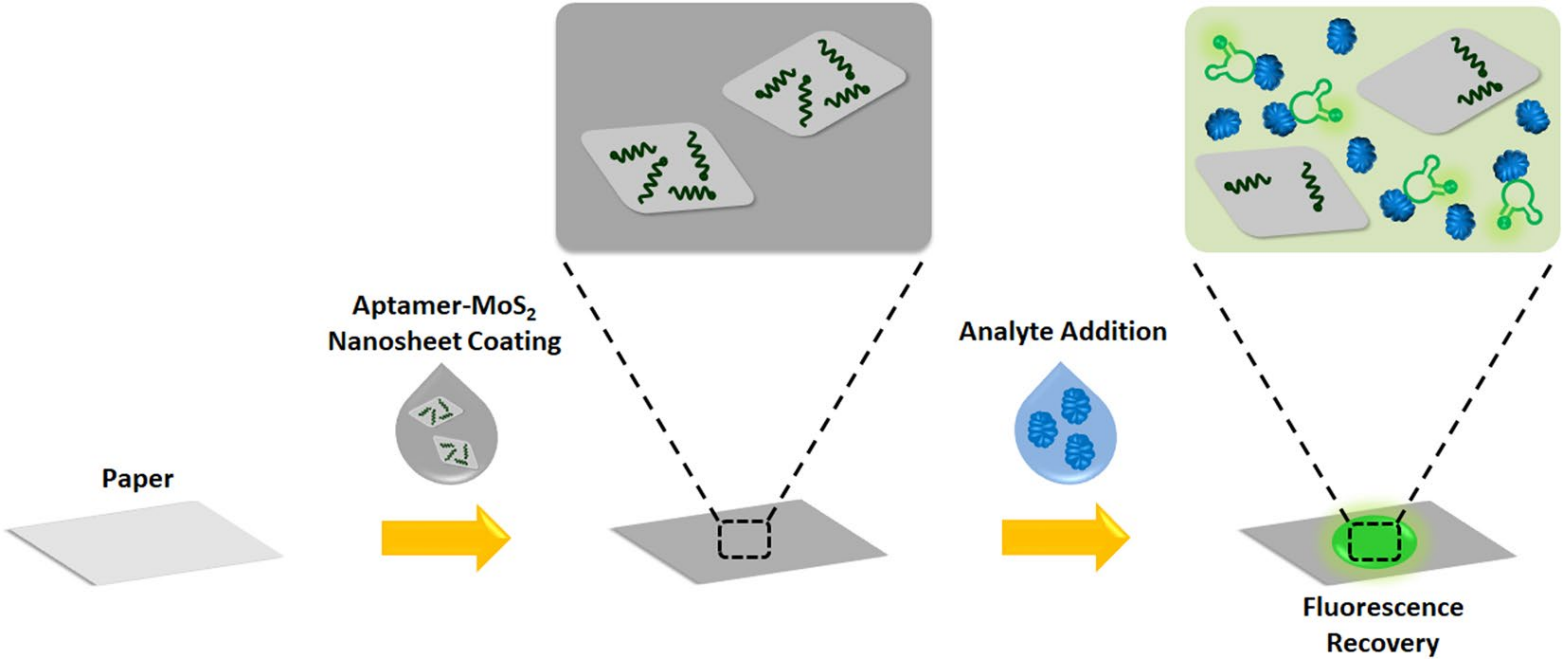
Paper-based MoS_2_ nanosheet-mediated FRET aptasensing process. Paper is first dipped in a solution containing fluorescently-labeled aptamers and MoS_2_ nanosheets, and then fully dried to form a test strip. In this state, the fluorescence of the aptamers on the test strip is quenched. When an analyte containing the targets of the aptamers is dropped onto the test strip, aptamers will be released from the nanosheet quenchers to bind to their targets, resulting in a measurable fluorescence recovery on the surface of the paper.

We translated a solution-based method for FRET-based aptasensing to a paper-based approach, as illustrated in Fig. 1. Fluorescently-labeled aptamers which bind to pLDH, as previously reported^25^, were incubated with fluorescence quenching nanomaterials to allow the aptamers to adsorb to the surface of nanomaterials, which quenches the fluorescence of the aptamers. MoS_2_ nanosheets, which were recently demonstrated to be viable quenching nanomaterials for solution-based aptasensing^19,26,27^, were selected as fluorescence quenchers. We have previously demonstrated that a solution-based FRET sensor using fluorescently-labeled aptamers and MoS_2_ nanosheets could detect pLDH with a limit of detection of 550 pM, indicating its high sensitivity^19^. To create a simpler and more portable sensor, a paper was dipped into the aptamer-quencher solution and fully dried to create a test strip, thus allowing the quenched aptamers to be deposited on the surface of the paper rather than being used in the less-portable solution form. Analytes could then be dropped onto the surface of the test strip to assess the presence or absence of target biomolecules. If an analyte contains the target of the aptamers (i.e., pLDH protein in this case), the target molecules will bind to the aptamers, inducing the aptamers to change their conformations and the target-aptamer complexes to be released from the nanomaterial quenchers. This will result in a distinct measurable fluorescence increase on the test strip.

**Figure 2:**
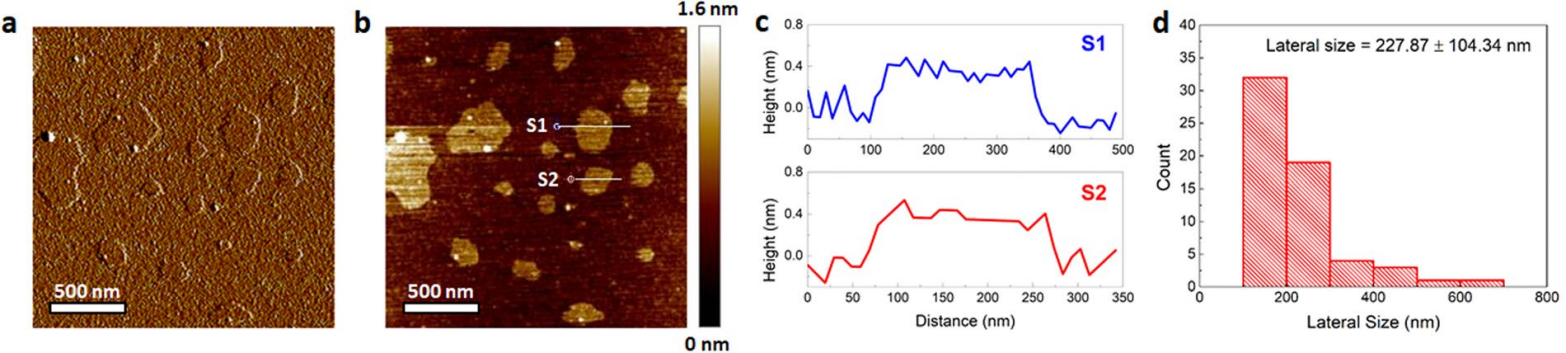
Surface morphological characterization of MoS_2_ nanosheets. (**a**) Representative 2D amplitude and (**b**) 2D height AFM images of MoS_2_ nanosheets. (**c**) Thicknesses of MoS_2_ nanosheets (i.e., S1 and S2 from (**b**)) are about 0.5 to 0.6 nm, indicating the single-layer thickness of the nanosheets. (**d**) Lateral size distribution of the nanosheets, as calculated from 60 nanosheet samples.

We first used tapping mode atomic force microscopy (AFM) to examine the surface morphology, thickness, and size distribution of our MoS_2_ nanosheets (Fig. 2). We observed that the nanosheets exhibited flat surfaces (Fig. 2a and b), with thicknesses of approximately 0.5 to 0.6 nm (Fig. 2c). This indicates that our MoS_2_ nanosheets are a single layer thick. In addition, the nanosheets had an average lateral size of 228 nm (Fig. 2d).

We then studied the use of both laboratory-grade and commercially-available papers for this FRET-based aptasensing scheme. Two brands of chromatography paper, the standard substrate for paper-based diagnostics, were used: Whatman Qualitative Filter Paper (No. 1) and Advantec Chromatography Paper (No. 51B). These two materials have similar listed properties and have both been used in paper diagnostics. Lens paper (VWR grade 541), a visibly thinner and more porous material, was tested as well. We also studied two types of common household products: A4 printer paper (Double A) and coffee filters (Boncafe). The paper grammage, a metric of density as measured in grams per square meter (gsm), varies widely among the different materials, according to the order of: lens paper (12 gsm) < coffee filter (approx. 22 gsm) < printer paper (80 gsm) < Advantec (87 gsm) ≈ Whatman (88 gsm).

**Figure 3:**
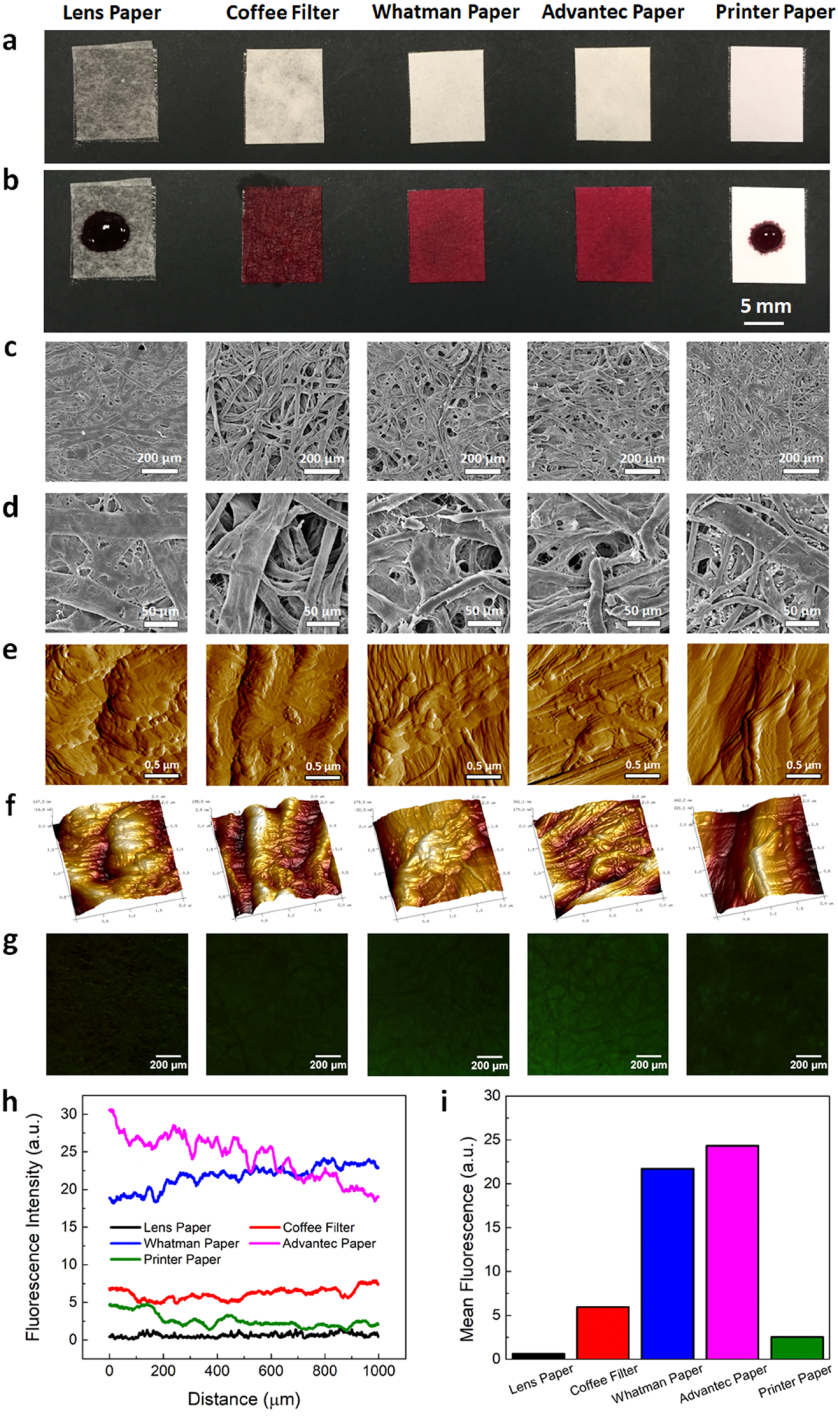
Characterization of the wettability, micro- and nanoscale surface morphologies, and aptamer distribution on different types of paper. (**a**,**b**) Surface wettability of different types of paper (i.e., lens paper, commercially-available coffee filter, two brands of chromatography filters (Whatman, Advantec), and A4 printer paper): (**a**) before and (**b**) immediately after the addition of a 30 µL droplet of food dye. The coffee filter and chromatography papers had high wettability as the dye permeated the paper immediately, while the lens and printer papers were less wettable, as the droplet retained its form on the paper surfaces. (**c**,**d**) Microscale morphology of different types of papers based on representative SEM images: (**c**) low and (**d**) high magnification images. (**e**,**f**) Nanoscale morphology of different types of paper based on representative AFM images: (**e**) 2D amplitude and (**f**) 3D topographical images showing the fibrous structure and nanoscale features of each type of paper. Scale bars represent 0.5 μm. (**g**) Fluorescence microscopy images showing the distribution of the fluorescently-labeled aptamers on different types of paper. All papers were dipped in 1 μM fluorescently-labeled aptamer solution and dried prior to imaging. All images have the same brightness and contrast to enable visual comparison. Scale bars represent 200 μm. (**h**) Fluorescence intensity profile as a function of distance and (**i**) mean fluorescence intensity of each type of aptamer-coated paper, as imaged in (**g**).

We started characterizing the hydrophilicity or hydrophobicity of the papers by assessing their wettability based on the spread of liquid across their surface. Pieces of each type of paper were mounted on a black substrate and the paper condition before and immediately after adding 30 µL of dye to the paper surface was recorded (Fig. 3a and b). We observed that the papers differ significantly in their wettability, as the dye spread instantaneously over the surface of the coffee filter and both the Whatman and Advantec chromatography papers, but remained as a droplet on the surface of the lens and printer papers (Fig. 3b). It is noteworthy that our sensing scheme, unlike many other paper diagnostics, does not require solution to wick across the paper to bring an analyte to different reaction zones. In fact, we quantified fluorescence recovery on test strips using fluorescence spectrometry, rather than imaging or visual inspection. As such, these fluorescence measurements can be taken from a very small area on the paper within the path of the reader. Thus, papers with higher wicking abilities, such as the coffee filter or chromatography paper, are not necessarily more suitable for use in the FRET aptasensor since the sample does not need to be spread across a large area on the test strip.

We next used scanning electron microscopy (SEM) and AFM to study the micro- and nanoscale surface morphologies of each type of paper, respectively (Fig. 3c to f). We observed that all papers had fibrous structures with height variations of a few hundred nanometers. The two brands of chromatography paper i.e., Whatman and Advantec, looked especially similar, with many thin wrinkles and fibers along their surfaces. Printer paper, on the other hand, possessed many nanoscale grains along its fibrous structure. Interestingly, these nanoscale grains were not observed from the other types of paper. Subsequently, we used fluorescence microscopy to study the distribution of aptamers throughout the microstructure of the paper (Fig. 3g to i). In a manner similar to the test strip preparation process, each type of paper was dipped in a solution of fluorescently-labeled pLDH aptamers and dried at room temperature. The fluorescence spread relatively uniformly over the area of the paper, indicating that the dipping process coated the paper evenly with aptamers, and that the aptamers remained on the paper and retained their fluorescence upon drying. On the coffee filter and chromatography papers, a darker network of fibers was visible and the aptamers appeared to fill the porous spaces of the paper between this network. Even with a high unquenched aptamer concentration of 1 µM and a 2-second exposure, the images had low fluorescence, demonstrating the challenge of using fluorescence imaging to analyze the test strip. Thus, for FRET sensing measurements, we chose to use a microplate reader to perform more quantitative assessments of the test strips.

**Figure 4:**
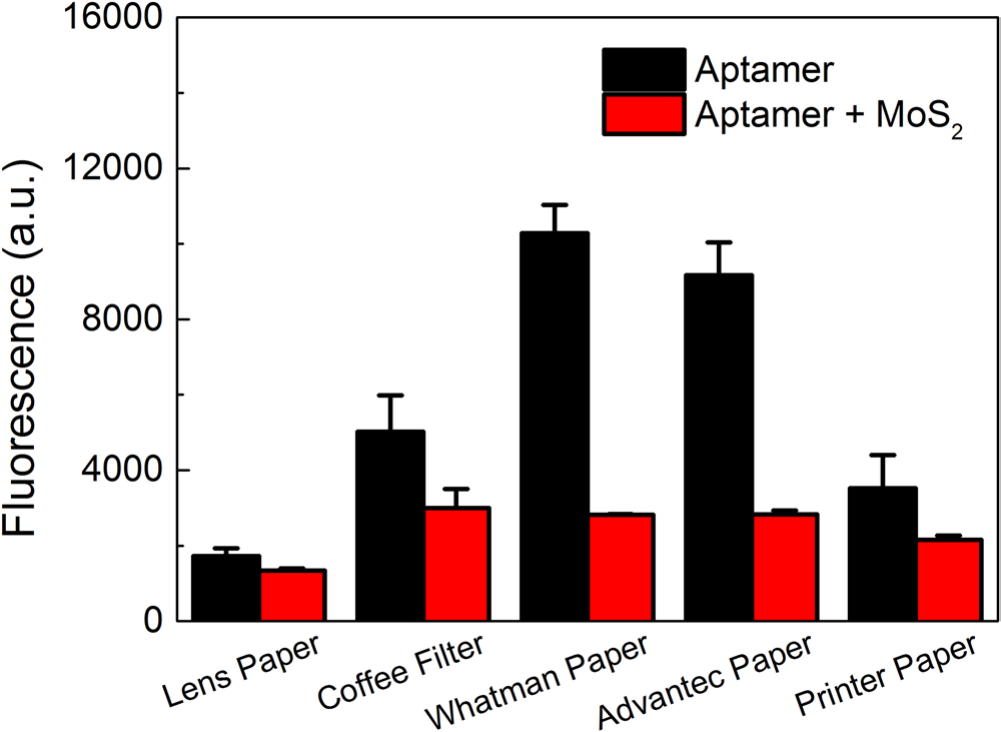
Fluorescence of free and quenched aptamers on test strips. Papers were coated with either 100 nM FAM-labeled pLDH aptamers (in black) or an incubation of 100 nM FAM-labeled pLDH aptamers and 25 µg/mL MoS_2_ nanosheets (in red). Fluorescence readings of the papers, after drying, were measured with excitation and emission wavelengths of 495 and 527 nm, respectively. For each type of paper, the test strip coated with the aptamer-nanosheet incubation was less fluorescent than the test strip coated with free aptamers, validating that aptamers remained quenched after the test strip was dried.

After imaging the distribution of free aptamers on the surface of each type of paper, we quantitatively compared the fluorescence of papers coated with free or quenched aptamers to ensure that fluorescence quenching can be measured from samples dried on paper, not just in solution (Fig. 4). Papers were dipped in either a solution of free fluorescently-labeled pLDH aptamers, or an incubation of fluorescently-labeled pLDH aptamers and MoS_2_ nanosheets, as was done to make test strips. After drying, the fluorescence of each paper was measured with a microplate reader. Despite using a tenfold lower concentration of aptamers (100 nM) than was used for the fluorescence imaging study in Fig. 3g to i, fluorescence was still detectable from each type of paper, demonstrating the value of using a fluorescence reader to analyze the FRET aptasensor with higher sensitivity. Interestingly, the fluorescence of the papers with free aptamers varied significantly across the different papers and was the highest on the Whatman and Advantec papers, followed by the coffee filter. In fact, this agrees with the results from Fig. 3g to i, where Whatman and Advantec papers also had higher fluorescence than other types of paper. In addition, it suggests that the papers with higher wettability, which include the coffee filter and chromatography papers, could absorb more aptamer solution and thus had higher fluorescence after drying. It is important to note that for all types of paper, the pieces coated with the aptamer-MoS_2_ incubation had lower fluorescence than the corresponding pieces coated with free aptamers. This demonstrated that the pLDH aptamers remained quenched by MoS_2_ nanosheets after the papers dried, and that the difference between the fluorescence of the quenched and free aptamers was distinguishable based on fluorescence readings of the papers.

After characterizing each type of paper and validating the use of a microplate reader to measure paper fluorescence, we investigated the performance of each type of test strip as an aptasensor for pLDH by monitoring the fluorescence recovery upon the addition of target or nonspecific proteins. Here, fibrinogen, globulin, and albumin were selected as nonspecific proteins because they are primary components of blood plasma and therefore, would be found in abundance in a blood sample. PBS was added to test strips as a negative control. Each type of paper was coated with an incubation of 100 nM pLDH aptamers and 25 µg/mL MoS_2_ nanosheets and dried, to create the starting test strips. 30 µL of either 1 µM pLDH, 1 µM of a nonspecific protein, or PBS was then dropped on top of the test strip, and the fluorescence of the strip was then monitored over the course of 30 min (Fig. S1a to Fig. S1e).

To account for differences in the initial fluorescence which might be caused either by random variations in the distribution of aptamers and quenchers on the test strip, or by small differences in the size or placement of the analyte droplet, the fluorescence measurements were normalized to the fluorescence value at t_0_, i.e., the reading which was taken immediately after an analyte addition. In a working aptasensor, the normalized fluorescence recovery induced by pLDH protein molecules should increase significantly above 1, indicating that the fluorescence of the sample increases as the fluorescently-labeled pLDH aptamers bind to their targets and are then released from the quenchers. Meanwhile, the normalized fluorescence recovery induced by nonspecific proteins should remain around 1, as nonspecific proteins should not interact with the aptamers and thus would not induce any fluorescence recovery.

On test strips of all types of paper, a 10–15% decrease in fluorescence was typically observed over a period of 30 min after the addition of nonspecific protein solutions or PBS. This fluorescence decrease occurred rather steadily (Fig. S1a to Fig. S1e) and resulted in a similar level of fluorescence for all nonspecific proteins after 30 min (Fig. S1f to Fig. S1j). Thus, analytes without the target pLDH protein molecules did not induce fluorescence recovery, which is an important requirement for sensor specificity. Though we expected the fluorescence signal to remain constant rather than slightly decrease after the addition of nonspecific proteins, a similar level of decline was generally observed from all nonspecific samples on a certain type of test paper, suggesting that this effect should not interfere with the sensing results.

**Figure 5:**
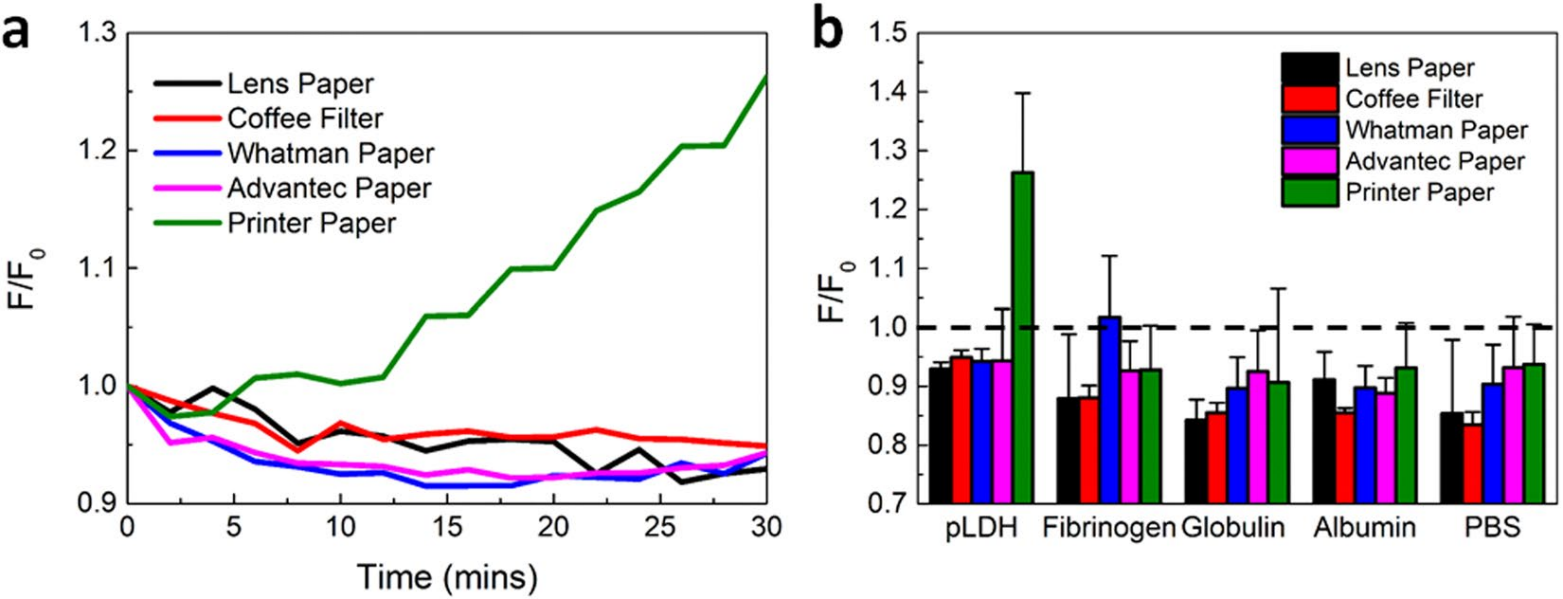
Fluorescence recovery measurements for all proteins and paper types. (**a**) Kinetics of the normalized fluorescence recovery induced by pLDH protein molecules on each paper test strip. A significant increase in fluorescence recovery could only be observed when pLDH was added to the printer paper test strip. (**b**) Normalized fluorescence recovery 30 min after sample addition, for all proteins and test strips. The normalized fluorescence recovery of pLDH on the printer paper test strip was notably higher than that for any other combination of protein and test strip.

Interestingly, we observed that pLDH protein molecules only induced a significant fluorescence recovery on the test strip made from printer paper, where fluorescence increased by over 25% after 30 min of incubation. This increase was distinct from the fluorescence response upon the addition of analytes lacking pLDH to the test strip, demonstrating the high specificity of the sensor (Fig. S1e and Fig. S1j). In fact, on all other types of paper test strips, pLDH did not induce a fluorescence increase and thus, it was indistinguishable from other nonspecific proteins, as the normalized fluorescence recovery remained below 1. The difference in the performance of different types of paper is shown in Fig. 5. The kinetics of pLDH-induced fluorescence recovery on all types of paper, as shown in Fig. 5a, demonstrated that specific fluorescence recovery occurred only on the printer paper test strip and not on test strips of other types of paper. A compilation of all fluorescence recovery results after 30 min also highlights the distinct fluorescence recovery induced by pLDH protein molecules on printer paper test strips (Fig. 5b).

Our results indicate that, unlike for many other paper-based diagnostics, materials with high wicking ability such as chromatography paper are not the most suitable substrates for our proposed FRET-based aptasensing mechanism. Here, printer paper was the only material which enabled successful FRET-based aptasensing. Our characterization data indicate that printer paper was unique from the coffee filter and chromatography papers because it was less wettable. Printer paper has also been reported to have a very small pore size of around 0.1 µm^28^. In contrast, Whatman No. 1 chromatography paper has 11 µm pores, and based on their appearance and low grammage, it is likely that lens paper and coffee filters have even larger pores. We propose that the printer paper enabled effective and specific paper-based FRET aptasensing because of its lower wettability and smaller pore size, which might prevent the displacement and loss of sensor components from the paper surface, thus facilitating more pLDH-aptamer binding events to induce a measurable fluorescence increase. In fact, Carrasquilla *et al*. have previously reported that small aptamers with a length of ~60 nucleotides are easily delocalized or washed out of Whatman No. 1 chromatography paper^22^. The pLDH aptamer sequence used in our study is only 36 nucleotides long. It is highly possible that pLDH aptamers are difficult to anchor on certain types of paper without the use of more advanced immobilization techniques, which would complicate sensor fabrication and increase production costs. As such, more cost-effective and commercially-available paper products should not be overlooked as potential materials for diagnostic assay development, since the materials used as the fundamental building blocks of a paper-based biosensor can greatly affect its sensing performance. In fact, this study has demonstrated that MoS_2_ nanosheets and aptamers for a disease biomarker could be simply adsorbed to printer paper to create a functional aptasensor for the facile and selective detection of infectious diseases such as malaria.

## Conclusions

In summary, we developed a facile and highly selective paper-based MoS_2_ nanosheet-mediated FRET aptasensor for rapid malaria diagnosis. We first investigated the performance of multiple types of paper, including both laboratory-grade and household products, as substrates for developing a FRET-based aptasensor. Both the product specifications and our characterization results indicated that the materials varied significantly in their wettability, pore size, and density. Consequently, these papers differed in their performance as substrates for FRET-based aptasensing. We then demonstrated successful and specific detection of the malarial biomarker pLDH, using a test strip made of aptamers and fluorescence-quenching MoS_2_ nanosheets adsorbed to a piece of printer paper. Fluorescence recovery, however, was not induced by pLDH on test strips made from lens paper, coffee filter paper, or chromatography paper. Our results highlight the importance of the rational design of paper-based diagnostic devices, and suggest that simple products such as printer paper should be investigated as more cost-effective and accessible alternatives to laboratory-grade materials when developing paper-based diagnostics. More complex assays could also benefit from a combination of different types of paper (for example, a more porous paper to filter out red blood cells could be connected to a less porous one to subsequently analyze the remaining blood plasma). We anticipate that this work will provide a further basis for the rational design and development of facile and highly selective paper-based diagnostics with optimized sensing performance.

## Experimental Section

### Materials

pLDH aptamers labeled with fluorescein (FAM) fluorescent dye at the 3′ end were purchased from Integrated DNA Technologies (with a sequence of 5′- GTT CGA TTG GAT TGT GCC GGA AGT GCT GGC TCG AAC - FAM - 3′). A pH 8 aptamer buffer with 20 mM Tris-HCl, 50 mM NaCl, 5 mM KCl, and 5 mM MgCl_2_ was used as the solvent. MoS_2_ nanosheets were prepared based on the electrochemical lithium-intercalation strategy, as reported previously^26,29^. All proteins were purchased from Sigma Aldrich and dissolved in 1 × PBS, with the exception of recombinant *falciparum* pLDH protein, which was purchased from Sino Biological and dissolved in water, based on the manufacturer’s instructions. The different types of paper used were lens paper (VWR grade 541), a commercially-available coffee filter (Boncafe), Whatman Grade 1 qualitative filter paper, Advantec chromatography paper No. 51B, and 80 gsm A4 printer paper (Double A).

### Paper characterization

The surface morphology of MoS_2_ nanosheets was first characterized using tapping mode AFM. The size distribution of these nanosheets was calculated by measuring the lateral size of 60 nanosheet samples. The surface wettability, nanoscale features, and aptamer uptake of each type of paper were then characterized. A 30 µL droplet of food dye was placed at the center of each piece of approximately 8 × 10 mm paper to characterize its surface wettability. Photographs of the papers were taken before and immediately after the addition of dye. The micro- and nanoscale morphologies of the surface of dry papers were examined using SEM (all paper samples were sputter-coated with a thin layer of gold before being imaged at magnifications of 80× and 300×) and tapping mode AFM (at a resolution of 512 × 512 points), respectively. To examine the distribution of aptamers on each type of paper, 30 µL of 1 µM FAM-labeled pLDH aptamers was dropped onto 8 × 10 mm pieces of each type of paper and then fully dried. Subsequently, the papers were taped flat on a glass slide and imaged using a fluorescence microscope (Nikon Eclipse TS100). All images were taken with 2 second exposure and 1× gain.

### Sensor preparation and fluorescence measurements

Test strips were made from approximately 8 × 10 mm pieces of paper, which were dipped into an incubation of 100 nM FAM-labeled pLDH aptamers and 25 µg/mL MoS_2_ nanosheets. Similarly-sized papers were coated with 100 nM FAM-labeled pLDH aptamers for the measurement of free aptamer fluorescence on paper. After fully drying, the coated papers were taped down inside a 12-well plate for fluorescence measurements. Fluorescence was measured using a Tecan M200 Infinite microplate reader. Readings were taken directly for the measurements of the fluorescence of dry papers. For fluorescence recovery experiments, 30 µL of 1 µM of either pLDH or a nonspecific protein solution was added to the center of the test strip and the fluorescence measurements of each well were taken every 2 min for 30 min. All fluorescence readings were measured with excitation and emission wavelengths of 495 and 527 nm, respectively, which were found to be the peak absorption and emission wavelengths of the FAM dye used to label each pLDH aptamer. Measurements were then normalized to the t_0_ fluorescence value measured immediately after the protein solution was added to the test strip, to account for variations in the original fluorescence of different test strips. All reported data is the average of 3 independent replicates.

## Electronic supplementary material

Supplementary Information
