# Supplementary material for: Paper-based MoS2 nanosheet-mediated FRET aptasensor for rapid malaria diagnosis
Source: Sci Rep. 2017 Dec 13;7:17510. doi: 10.1038/s41598-017-17616-3 (PMC5727500; doi:10.1038/s41598-017-17616-3)
Supplement: Supplementary file 1 — Supplementary Information [file 41598_2017_17616_MOESM1_ESM.doc]

**Electronic Supplementary Information**

**Paper-based MoS_2_ nanosheet-mediated FRET aptasensor for rapid malaria diagnosis**

**Alisha Geldert^1, †^, Kenry^1,2,3, †^, Chwee Teck Lim^1,2,3,4,*^**

^1^Department of Biomedical Engineering, National University of Singapore, Singapore 117576

^2^NUS Graduate School for Integrative Sciences and Engineering, National University of Singapore, Singapore 117456

^3^Centre for Advanced 2D Materials and Graphene Research Centre, National University of Singapore, Singapore 117543

^4^Mechanobiology Institute, National University of Singapore, Singapore 117411

^†^These authors contributed equally to the work.

*Correspondence

Chwee Teck Lim ([ctlim@nus.edu.sg](mailto:ctlim@nus.edu.sg))

Department of Biomedical Engineering

National University of Singapore

9 Engineering Drive 1

Singapore 117576, Singapore

**KEYWORDS:** Paper-based diagnostics; Aptamer; FRET; Plasmodium lactate dehydrogenase (pLDH)

**
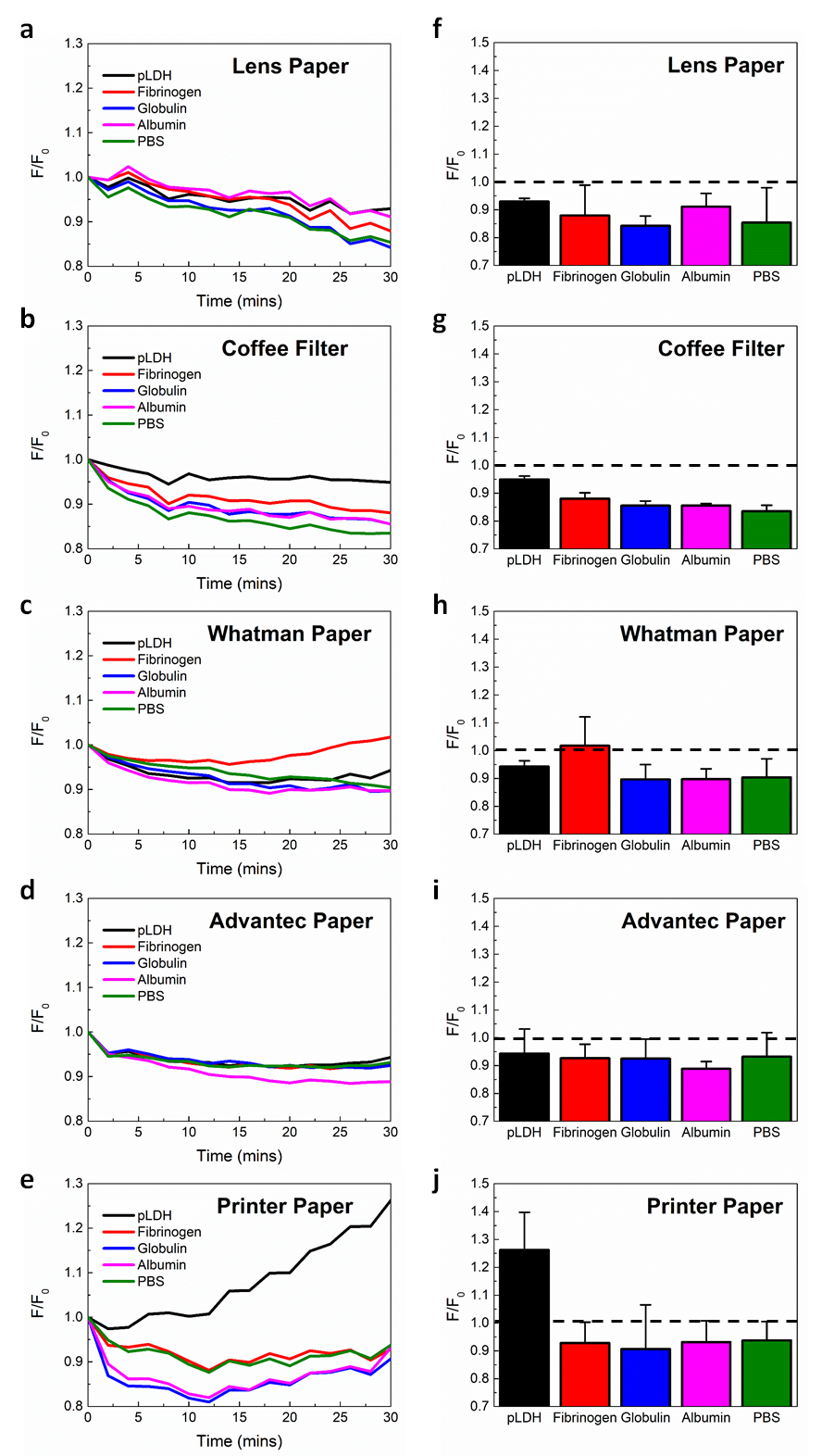
**

**Figure S1 | Performance of FRET-based aptasensing of pLDH on different types of paper.** 30 µL of 1 µM pLDH protein or nonspecific protein was placed on top of test strips dried with a mixture of 100 nM FAM-labeled pLDH aptamer and 25 µg/mL MoS_2_ nanosheets. (a-e) Fluorescence recovery, normalized to the fluorescence measured immediately after sample addition, was tracked over 30 min. The fluorescence of the (a) lens paper, (b) coffee filter, (c) Whatman paper, and (d) Advantec paper test strips remained constant or decreased, regardless of the protein added. However, on (e) printer paper test strips, pLDH induced a steady fluorescence increase over 30 min while all nonspecific proteins had negligible effect. (f-j) Normalized fluorescence recovery induced by each protein 30 min after being added to the test strips. Only on the (j) printer paper test strip did pLDH induce a significant fluorescence increase (with a normalized fluorescence recovery above 1), making it differentiable from nonspecific proteins. All fluorescence readings were measured with excitation and emission wavelengths of 495 and 527 nm, respectively.
